# The Role of Calcium/Calcium-Dependent Protein Kinases Signal Pathway in Pollen Tube Growth

**DOI:** 10.3389/fpls.2021.633293

**Published:** 2021-03-09

**Authors:** Hao Yang, Chen You, Shaoyu Yang, Yuping Zhang, Fan Yang, Xue Li, Ning Chen, Yanmin Luo, Xiuli Hu

**Affiliations:** ^1^State Key Laboratory of Wheat & Maize Crop Science, Henan Agricultural University, Zhengzhou, China; ^2^College of Life Science, Henan Normal University, Xinxiang, China; ^3^Department of Biology, Taiyuan Normal University, Jinzhong, China

**Keywords:** calcium, calcium-dependent protein kinases, pollen tube, plant, signaling

## Abstract

Pollen tube (PT) growth as a key step for successful fertilization is essential for angiosperm survival and especially vital for grain yield in cereals. The process of PT growth is regulated by many complex and delicate signaling pathways. Among them, the calcium/calcium-dependent protein kinases (Ca^2+^/CPKs) signal pathway has become one research focus, as Ca^2+^ ion is a well-known essential signal molecule for PT growth, which can be instantly sensed and transduced by CPKs to control myriad biological processes. In this review, we summarize the recent progress in understanding the Ca^2+^/CPKs signal pathway governing PT growth. We also discuss how this pathway regulates PT growth and how reactive oxygen species (ROS) and cyclic nucleotide are integrated by Ca^2+^ signaling networks.

## Introduction

The calcium ion (Ca^2+^), as a central second messenger in plants, coordinates a variety of physiological responses by binding the calcium sensors, which decode the calcium signatures and elicit different cellular responses. In plants, there are four main classes of calcium sensors: calmodulin (CaM) or CaM-like proteins (CMLs), calcineurin B-like proteins (CBLs), CBL interacting protein kinases (CIPKs), and the calcium-dependent protein kinases (CPKs) and their relatives, CDPK-related kinases (CRKs; Harper et al., 2004; Dodd et al., 2010). Among them, CPKs share the unique feature of combining the calcium-binding motifs and protein kinase domain (PKD) on the same peptide. CPKs are implicated in the regulation of plant development, as well as in biotic and abiotic stress signaling. The different tissue- and developmental-stage expressions of the CPKs possess specific functions; for example, *At*CPK28 and *At*CPK3/4/6/11 have roles in shoot and root development, respectively, and *At*CPK6/33 may be involved in the regulation of floral transition (see the review by Yip Delormel and Boudsocq, 2019). Significantly, a number of *At*CDPKs are mainly expressed in pollen, indicating their involvement in pollen development and/or pollen tube (PT) growth, which is crucial for sexual reproduction in flowering plants. Successful fertilization begins with pollen grains landing on the stigma and germination of the PT. Upon pollen landing on the stigma, the PT rapidly elongates and penetrates the transmitting tract to deliver the immotile sperm to the ovule for double fertilization (Higashiyama and Yang, 2017). During this process, Ca^2+^ is well-known to control pollen germination, PT growth, and intercellular communication between PT and female tissue (Ge et al., 2007; Zheng et al., 2019). However, we do not fully understand how these specific Ca^2+^/CPKs signal pathways regulate PT growth. In this review, we summarize the key findings of the Ca^2+^/CPKs signaling pathway in PT growth and further address the interrelationship between Ca^2+^ signaling with other complex signaling networks such as reactive oxygen species (ROS) and cyclic nucleotide.

## Composition and Construction of Pollen Tube

The PT is a tubular structure that germinates from the aperture in pollen. In angiosperms, the cell wall of the PT usually comprises two layers: the outer fibrillar layer that is mainly composed of pectin, hemicellulose, and cellulose, and the inner layer of callose (Taylor and Hepler, 1997). The tip of the PT comprises a single pectin layer, which is the most elastic region and the expansion point of PT growth. Some studies indicated that inhibition of cellulose biosynthesis can affect the morphology and structural integrity of Petunia and Lily PTs (Anderson et al., 2002), while pectin that is synthesized in the Golgi apparatus and then secreted into the cell wall by exocytosis can strengthen the mechanical strength and ductility of the PT (Li et al., 1994; Hasegawa et al., 1998). Interestingly, callose is only deposited on the inner layer of the cell wall of the PT, except for the tip, and it also has a role in the correct recognition of pollen and stigma (Lush and Clarke, 1997; Dearnaley et al., 1999; Kuboyama and Takeda, 2000). Further, some glycoproteins are deposited in the PT, i.e., arabinogalactan proteins (AGPs) and lipid transfer protein 5 (LTP5; Cheung et al., 1995; Chae et al., 2009).

**Figure 1:**
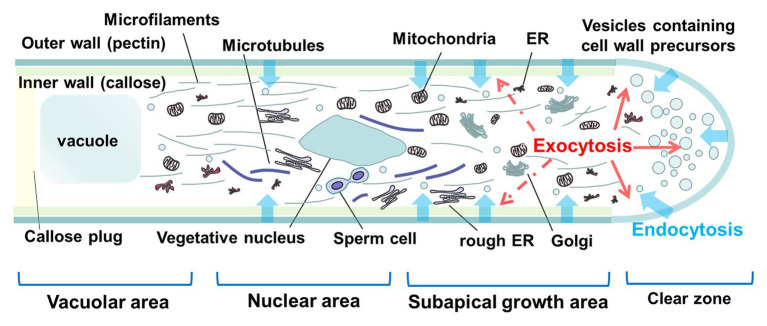
Diagrammatic representation of the structure of the tip region of a pollen tube (PT). The tip of a PT can be classically divided into four zones from the tip to the base: clear zone, subapical growth area, nuclear area, and vacuolar area. The bulk of exocytosis occurs in the apical region as shown with the solid red arrows, while certain exocytic vesicles may also be secreted to the subapical growth area, as shown with the dotted arrows. Endocytosis can occur in the apical and subapical growth areas and nuclear area as shown with the blue arrows (ER is endoplasmic reticulum).

The structure of the PT can be divided into four different zones according to Franklin’s description (Figure 1; Franklin-Tong, 1999). At the extreme tip of the PT, a “clear zone” is filled with secretory vesicles that package many cell wall components, which will then be incorporated into the apical dome of the PT tip for elongation; behind the “clear zone” is a subapical growth area, which contains most of the cytoplasm and organelles such as mitochondria, Golgi complexes, endoplasmic reticulum (ER), and cytoskeletal components. At the bottom of the germinated PT are the vacuolar area and the cell nuclear area, which contain the vegetative nucleus and generative (or sperm) cell. The formation of vacuoles maintains tube turgor and pushes the cytoplasm to the apex of the PT. To restrict the cytoplasm to the apical region of the growing tube, a series of callose plugs are formed at regular intervals behind the tip. Although the composition and construction of the PT are well understood, the mechanics of its elongation are unclear. In addition to the conventional hydrodynamic model, ion dynamics and the cell wall model (Zonia and Munnik, 2011; Liu and Hussey, 2014), a new Hechtian model of PT tip growth has been put forward (Lamport et al., 2017). Briefly, the new model proposes that a viscoplastic pectic cell wall is mechanically coupled to the plasma membrane by Hechtian adhesion, which transmits wall strain to the plasma membrane and thus regulates Ca^2+^ and other ion fluxes that regulate the exocytosis of wall precursors. Moreover, the “kiss-and-run” mode of exocytosis/endocytosis at the PT apex and “durotropic” movement, namely exhibiting increased movement speed in stiffer materials, have also been proposed and challenge some previous standpoints about PT elongation (see the review by Adhikari et al., 2020). In any case, all the models mentioned above involve Ca^2+^ as a key signal.

## The Role of Ca^2+^ in Pollen Tube Growth

It is well established that a tip-focused calcium gradient is essential for pollen germination and PT growth (Holdaway-Clarke et al., 2003; Iwano et al., 2009; Michard et al., 2009; Steinhorst and Kudla, 2013). The elevation of the Ca^2+^ gradient is correlated with pulsed tube growth. Some studies indicated that Ca^2+^ can directly affect turgor formation during PT growth by affecting the formation of the vacuole (Li et al., 2017). Additionally, asymmetric Ca^2+^ accumulation within the tip is associated with reorientation of growth in that direction (Malho and Trewavas, 1996). In most studies, [Ca^2+^]_cyt_ oscillations correlate with oscillations of PT growth speed (Holdaway-Clarke et al., 1997). Channels that account for oscillatory Ca^2+^ influx across the plasmalemma mainly include the stretch-activated Ca^2+^ channels (SACs), the cyclic nucleotide-gated channels (CNGCs; Frietsch et al., 2007), and the glutamate receptor-related channels (GLRs; see the review by Hepler et al., 2012). SACs locate at the extreme apex of the PT and open in response to deformation of the plasma membrane caused by PT growth (Dutta and Robinson, 2004). In *Arabidopsis*, two SACs, namely MCA1 and MCA2, have been identified in the root; however, it remains unknown if related proteins function in PTs (Nakagawa et al., 2007). In addition to the first identified and molecularly characterized CNGC18, there are five additional CNGCs (namely CNGC7, 8, 9, 10, and 16) that are potentially relevant for pollen ion fluxes (Tunc-Ozdemir et al., 2013; Gao et al., 2016). A recent study indicates that CNGC18/8/7 together with calmodulin 2 (CaM2) constitutes a molecular switch that either opens or closes the calcium channel, depending on [Ca^2+^]_cyt_ levels during PT growth (Pan et al., 2019). Subsequently, a breakthrough study uncovered that MILDEW RESISTANCE LOCUS-O (MLO) proteins can regulate PT guidance in response to ovular signals by recruiting the CNGC18 to the plasma membrane in order to modify Ca^2+^ gradients in the growing PT (Meng et al., 2020). Among all known CNGCs, only *At*CNGC18 and *Os*CNGC13 are reported to be highly expressed in the pistils and to act as a novel maternal sporophytic factor required for PT guidance (Gao et al., 2016; Xu et al., 2017). By pharmacology, loss-of-function, and heterologous complementary approaches, some studies indicate that GLRs facilitate Ca^2+^ influx, modulating the apical [Ca^2+^]_cyt_ gradient and consequently the impact on PT growth (Qi et al., 2006; Michard et al., 2011; Vincill et al., 2012). Interestingly, *At*GLRs are inactive when expressed alone in *Xenopus* oocytes, implying that GLRs may be subject to a plant-specific activation mechanism by CPKs (Roy et al., 2008; Alfieri et al., 2020). A recent study revealed that CORNICHON HOMOLOG (CNIH) proteins are essential for sorting, trafficking, and localizing *At*GLRs (Wudick et al., 2018). More importantly, the result of coexpressing *At*CNIH4 or *At*CNIH1/4 with a PT expressed *At*GLR3.3 or *At*GLR3.2 in COS-7 cells further confirms that CNIH proteins can enhance *At*GLR channel activity, and with binding specificity (Wudick et al., 2018). In ovules, 1-aminocyclopropane-1-carboxylic acid (ACC), a precursor of ethylene synthesis, stimulates GLR-dependent Ca^2+^ elevation, which in turn promotes LURE1 secretion and PT attraction (Mou et al., 2020). And whether ACC can also act as the most potent elicitor of GLR-mediated Ca^2+^ elevations in PT requires further study. In addition to these plasma membrane located Ca^2+^ channels, some internal Ca^2+^ channels located at a vacuole or endoplasmic reticula, such as ACA2/7/8/9/10 and ECA1, are responsible for fine-tuning the Ca^2+^ gradient by sequestration of the ion (Harper et al., 1998; Hwang et al., 2000; Iwano et al., 2009; Lucca and León, 2011; Michard et al., 2017; Li et al., 2018a). In addition to being a signal molecule, the Ca^2+^ ion is also required for cross-linking cell wall components. At the extreme apex of a growing PT, methyl-pectin is secreted as the main new cell wall material, which forms rather loose ionic bonds with Ca^2+^, resulting in reduced cell wall rigidity. As soon as [Ca^2+^]_cyt_ increases, the pectin methylesterase is transported to the apex by exocytosis, resulting in de-methoxylation of methyl-pectin and cross-linking with free Ca^2+^, which increases cell wall rigidity (Bosch and Hepler, 2005). In the process of cell wall remodeling, a self-regulatory network modulating oscillatory growth cycles of an elongating PT also integrates changes in the concentration of [Ca^2+^]_cyt_, apical exocytosis of methyl-pectin and pectin methyl esterase (PME), and regulation of SACs, as well as the contribution of F-actin and ROP1 signaling (see the review by Steinhorst and Kudla, 2013).

## The Structure and Functions of Ca^2+^ Signal Decoder CPKs

**Figure 2:**
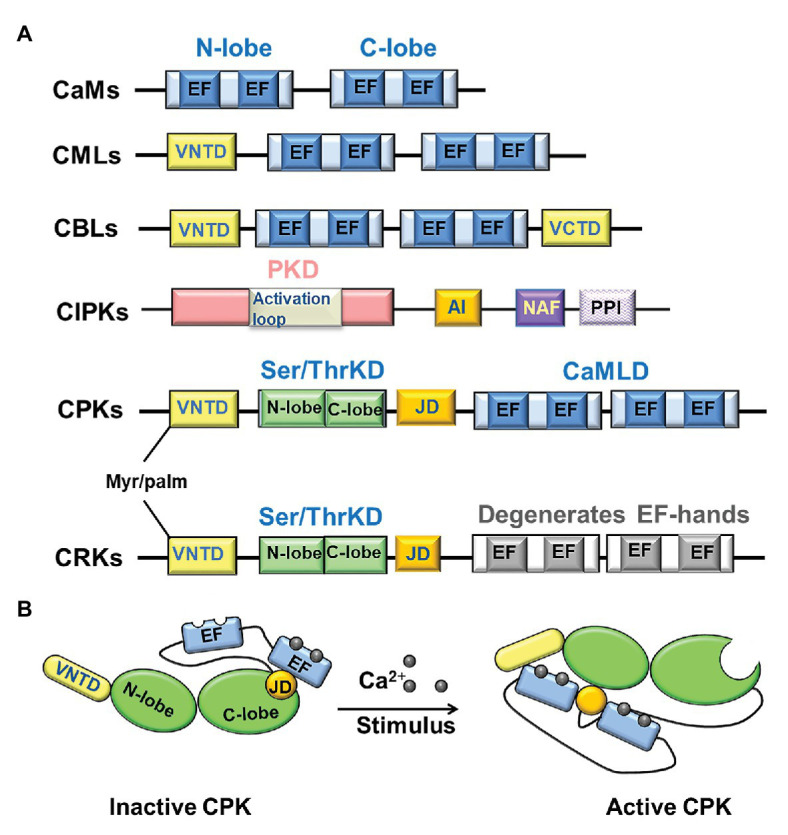
The structures of four calcium sensors and the activation mechanism of calcium-dependent protein kinases (CPKs) by calcium-binding. **(A)** Domain organization of the four main classes of calcium ion (Ca^2+^) sensors in plants. **(B)** Activation mechanism of CPKs by calcium-binding. When Ca^2+^ concentration is low, the C-lobe is loaded with Ca^2+^ and interacts with the junction domain (JD), forming an inactive conformation, which blocks the kinase domain access to the substrate. While Ca^2+^ concentration is elevating, the N-lobe binds Ca^2+^, resulting in an active conformation, which drives the auto-inhibitory JD out of the active site. EF: EF-hand usually occurs in pairs, N-lobe having a lower calcium affinity than the C-lobe; PKD: protein kinase domain; NAF: asparagine-alanine-phenylalanine domain mediating interaction with the calcineurin B-like proteins (CBLs); PPI: protein-phosphatase interaction domain mediating the interactions of CIPK with 2C-type protein phosphatases (PP2Cs); VNTD: variable N-terminal domain, where the myristoylation (myr) and palmitoylation (palm) occur; VCTD: variable C-terminal domain; JD/AI: an auto-inhibitory junction domain; CaMLD: a C-terminal CaM-like domain classically with 4 EF-hands Ca^2+^-binding motifs.

The Ca^2+^ signal can be decoded and relayed by a series of phosphorylation cascades mainly regulated by four families of protein kinases (Harper et al., 2004). Among them, CPKs can be directly activated by Ca^2+^ and phosphorylate downstream effectors to regulate myriad biological processes. The representative structure of CPKs harbors a variable N-terminal domain (VNTD) followed by a PKD and an auto-inhibitory junction domain (JD) that is linked to the C-terminal calmodulin-like domain (CaMLD) with EF-hand Ca^2+^-binding sites (Harper et al., 1991; Cheng et al., 2002; Hrabak et al., 2003). The VNTD is important not only for membrane localization when modified by palmitoylation and myristoylation at the cysteine and glycine residues, respectively; it is also essential for specific interaction with targets (Stael et al., 2011; Boudsocq and Sheen, 2013). The JD serves as a pseudosubstrate that blocks the kinase active center in the absence of Ca^2+^ and releases autoinhibition upon Ca^2+^ binding to EF-hands within the CaMLD domain (Figure 2; Harmon et al., 1994; Liese and Romeis, 2013). CPKs have been identified throughout the plant kingdom and constitute a large multigene family in various plant species, i.e., 34 CPKs identified in *Arabidopsis thaliana*, 29 CPKs identified in *Oryza sativa*, and 32 CPKs identified in *Zea mays* (Cheng et al., 2002; Asano et al., 2005; Khalid et al., 2019). The CPK superfamily members have been implicated in many biological processes, such as development, metabolism, and biotic and abiotic stress responses (reviewed in Klimecka and Muszynska, 2007; Asano et al., 2012). Given the huge number of CPKs with specific functions in different cells or tissue, one important question is how the Ca^2+^/CPKs signal pathway regulates pollen germination and PT growth.

## The Role of CPKs in Pollen Tube Growth

**Figure 3:**
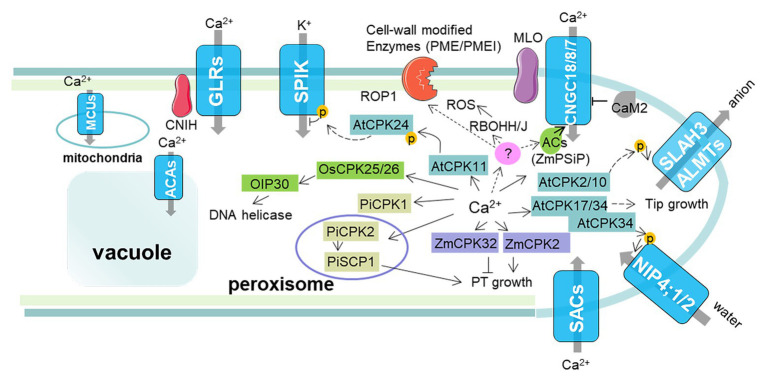
The proposed Ca^2+^/CPK signaling regulating PT growth. On the plasmalemma of the PT, calcium entry mainly occurs through three different channels: the stretch-activated Ca^2+^ channels (SACs), the cyclic nucleotide gated channels (CNGCs), and the glutamate receptor-related channels (GLRs). CNGCs become activated by the binding of adenosine 3', 5'-cyclic monophosphate (cAMP), which are produced by adenylate cyclases (ACs) and inhibited by calmodulin (CaM) binding. Moreover, MLO5/9 can recruit the CNGC18 with asymmetric distribution and result in a change in PT growth direction. The SACs are located at the extreme apex of the tube in response to the deformation of the plasma membrane caused by growth. GLRs as a ligand Ca^2+^ gated channel are transported, targeted, and activated by CORNICHON HOMOLOG (CNIH) proteins. Some other Ca^2+^ channels located on the organelle membrane are also involved in fine-tuning of the cytoplasmic Ca^2+^ concentration and affecting the PT growth, such as mitochondrial calcium uniporters (MCUs) and Ca^2+^-ATPases (ACAs). Ca^2+^ signals are perceived by CPKs that decode the information presented in specific Ca^2+^ signatures and regulate PT growth. In *Arabidopsis*, the Ca^2+^/AtCPK11 signal pathway phosphorylates *At*CPK24, which will further phosphorylate the K^+^ influx channel SPIK, resulting in the inhibition of PT elongation. The Ca^2+^/*At*CPK2/10 signal pathway phosphorylates the anion channel SLAH3 and some ALMTs to export anion at the PT tip. Ca^2+^/*At*CPK17/34 can promote pollen tip growth and tropism. The Ca^2+^/*At*CPK34 signal pathway can phosphorylate pollen-specific aquaporins NIP4;1 and NIP4;2 to ensure pollen germination and PT growth. In maize, Ca^2+^/*Zm*CPK20 positively regulates PT growth, while Ca^2+^/*Zm*CPK32 negatively regulates PT growth. In petunias, *Pi*CPK2 can interact with the small CDPK-interacting protein 1 *Pi*SCP1 to affect PT growth, presumably by mediating peroxisome function, while *Pi*CPK1, which is localized in the plasma membrane, can regulate the polarity of PT growth. In rice, Ca^2+^/*Os*CPK25/26 can phosphorylate DNA helicase OIP30 in mature pollen. Moreover, the Ca^2+^/CPK signal may also integrate and coordinate with other signaling systems, such as ROP1 signaling, reactive oxygen species (ROS), and cAMP.

Calcium-dependent protein kinases, as the vital components in Ca^2+^ signaling pathways, have been implicated in many aspects of plant life including development and abiotic and biotic stress responses (Simeunovic et al., 2016). The first CPK found to be involved in pollen germination and PT growth was in maize, as the inhibition of this pollen-specific CPK (*Zm*CPK20) impaired both the pollen germination and growth (Estruch et al., 1994; Moutinho et al., 1998). Further analysis of the expression patterns of *ZmCPKs* using the maize gene expression atlas revealed that about 12 *ZmCPKs* were predominantly accumulated in the anther (Stelpflug et al., 2016; Li et al., 2018a). Some proteome studies also found that many CPKs accumulated in maize pollen and many phosphorylate specific substrates upon PT germination and growth. The crucial role of the maize CDPK in PT growth is further substantiated by the function study of *Zm*CPK32 (Li et al., 2018b). In contrast to most CPKs’ positive regulation of PT growth, ZmCPK32 as a pollen-specific CPK was demonstrated to negatively regulate the PT growth, as a transient expression of *ZmCPK32* in tobacco *via* microparticle bombardment suppressed both the PT germination and growth (Li et al., 2018a). In *Petunia inflate*, *Pi*CPK1 and *Pi*CPK2 were highly expressed in PT and had distinct functions. The *Pi*CPK2 is involved in PT extension by mediating peroxisome function in conjunction with a small CDPK-interacting protein 1 (*Pi*SCP1; Guo et al., 2013), while the *Pi*CPK1 is likely a key regulator of growth polarity by regulating Ca^2+^ homeostasis (Yoon et al., 2006). Moreover, five of the 34 CDPK isoforms in the *Arabidopsis* are highly expressed in pollen, including *AtCPK14*, *16*, *17*, *24*, and *34* (Harper et al., 2004). Among them, the genetic evidence indicated that *AtCPK17* and *AtCPK34* are essential for PT growth in response to a Ca^2+^ signal in the apical dome (Figure 3; Myers et al., 2009). How *AtCPK17* and *AtCPK34* influence the PT polarized tip growth remains poorly understood, and whether *At*CPK17/34 has a regulatory function in the rho-GTPase of plants (ROP) pathway awaits further confirmation (Yang and Fu, 2007; Zhou et al., 2009). Two pollen-specific aquaporins, *At*NIP4;1 and *At*NIP4;2, were identified, which can be phosphorylated by *At*CPK34 *in vitro* and have a role in pollen germination and PT growth (Di Giorgio et al., 2016). *At*CPK2, *At*CPK20, and *At*CPK6 were shown to promote PT growth by activating the anion channel SLAH3 and ALMT12/13/14 at the pollen tip (Gutermuth et al., 2013, 2018). K^+^ influx into PT is also essential for PT growth. Further studies showed that *At*CPK11 and *At*CPK24 (the closest homolog of *Zm*CPK32) negatively affect PT elongation by mediating the Ca^2+^-dependent inhibition of the inwardly rectifying K^+^ channels (Zhao et al., 2013). In *O. sativa*, *Os*CPK21 plays an essential role in pollengenesis, possibly *via* indirectly regulating the transcription of MIKC*-type MADS box proteins (Wen et al., 2019). Moreover, *Os*CPK25/26 can phosphorylate the predominantly pollen-expressed OIP30 (a RuvB-like DNA helicase 2) and likely affect pollen development by transcriptional control of gene expression (Wang et al., 2011).

## How Ca^2+^/CPKs Regulates the Pollen Tube Growth

Although mounting evidence indicates that the Ca^2+^/CPKs signal pathway has a role in PT growth, how it regulates PT growth is still unclear. The key to unlocking the underlying mechanisms depends on the identification of downstream signal pathway targets. Simeunovic et al. (2016) have comprehensively summarized the identified CPKs targets in plants, while only a small number of CPKs targets have been identified in pollen, mainly including some ion channels (or aquaporin) such as *At*SPIK, *At*SLAH3, *At*ACA8, and *At*NIP4;1/2 (see Table 1). The activity of PT specific shaker pollen inward K^+^ channel (SPIK) was inhibited by *At*CPK24, which is phosphorylated and activated by *At*CPK11 (Figure 3; Zhao et al., 2013). Disruption of SPIK will reduce K^+^ influx and impair pollen germination and PT growth (Mouline et al., 2002). Moreover, the Ca^2+^/CPKs signal pathway to control PT growth *via* anion channel (*At*SLAH3 and ALMT12/13/14) activation is confirmed by reverse genetics and electrophysiology (Figure 3; Gutermuth et al., 2018). The tip-focused Ca^2+^ gradient is essential for PT growth, which requires Ca^2+^ channel distributions in PT. The cyclic nucleotide-gated channel 18 (CNGC18) is functionally validated for Ca^2+^ influx across the plasma membrane of PT. Some research reveals a potential feed-forward mechanism in which CPK32 activates CNGC18, further promoting calcium entry during the elevation phase of Ca^2+^ oscillations in the polar growth of PTs (Zhou et al., 2014). And whether the activities of other CNGCs are directly and indirectly influenced by Ca^2+^/CPKs requires further investigation. *At*ACA8, a Ca^2+^-ATPases to extrude Ca^2+^ to the apoplast, is confirmed to be phosphorylated by *At*CPK16 *in vitro* (Giacometti et al., 2012). All these results suggest that the Ca^2+^/CPKs signal pathway may regulate PT growth by maintaining the appropriate intracellular ion concentrations at the apex *via* fine-tuned diversified ion channels. Besides these, the Ca^2+^/CPKs signal pathway may crosstalk with other signal molecules such as ROS, which are generated by respiratory burst oxidase homolog (Rboh) NADPH oxidases and also involved in PT growth. In *Arabidopsis*, RBOHH and RBOHJ were revealed to not only slow down PT growth but also maintain PT integrity when regulated by the RALF-BUPS/ANX complex (Boisson-Dernier et al., 2013). The direct regulation of RBOHD activity by Ca^2+^/CPKs has been reported in *Arabidopsis* and Potato (Kobayashi et al., 2007; Liu and He, 2016). However, whether there are some specific CPKs in pollen that are responsive to phosphorylate Rboh remains unknown so far. Moreover, cell wall-modifying enzymes are crucial for PT growth. Some studies show that PME and PME inhibitor (PMEI) modulate the rapid growth of the PT (Röckel et al., 2008). It will be interesting to explore these enzymes, which are potential downstream targets of the Ca^2+^/CPKs signal pathway. We also summarized a model to illustrate the Ca^2+^/CPKs signal pathway regulating PT growth.

**Table 1:** Overview of the identified CPKs in pollen.

Name*^a^*	Gene ID*^b^*	Location*^c^*	Targets*^d^*	Physiological relevance*^e^*	References*^f^*
***Arabidopsis thaliana***					
*At*CPK2	AT3G10660	ER, MB	*At*RBOHD/F (PM), *At*SLAH3 (PM), ALMT12/13/14 (PM)	Reduced ROS production in *cpk1,2* double mutants; reduced anion currents and fluxes are reduced in *cpk2,20* double mutants	Lu and Hrabak, 2002; Harper et al., 2004; Gao et al., 2013; Gutermuth et al., 2013, 2018
*At*CPK14	AT2G41860	MB	-	-	Harper et al., 2004
*At*CPK16	AT2G17890	PM	ACA8 (PM), *At*Di19-2 (N, C)	-	Curran et al., 2011; Giacometti et al., 2012
*At*CPK17	AT5G12180	PM	-	Reduced pollen transmission efficiency in *cpk17/34* double mutants	Myers et al., 2009
*At*CPK20	AT2G38910	S, MB	*At*SLAH3 (PM), ALMT12/13/14 (PM)	Anion currents and fluxes are reduced in *cpk2,20* double mutants	Gutermuth et al., 2013, 2018
*At*CPK24	AT2G31500	PM, N	SPIK,14-3-3	Impairing the Ca^2+^-dependent inhibition of K^+^ in currents and PT elongation	Zhao et al., 2013; Swatek et al., 2014
*At*CPK26	AT4G38230	N, C	-	-	Harper et al., 2004
*At*CPK34	AT5G19360	PM	NIP4;1/2	Fewer seeds per silique and reduced pollen germination and PT length in *nip4;1/2* mutant	Di Giorgio et al., 2016
***Zea mays***					
*Zm*CPK20	GRMZM2G365815	-	-	-	Estruch et al., 1994
*Zm*CPK32	GRMZM2G332660	PM	-	Inhibition of PT growth by transient expression of *ZmCPK32* in tobacco pollen	Li et al., 2018a
***Petunia inflate***					
*Pi*CPK1	DQ147913	PM	*Pi*SCP1	Loss of growth polarity Inhibited pollen germination and tube growth	Yoon et al., 2006; Guo et al., 2013
*Pi*CPK2	DQ147912	P	*Pi*SCP1	Inhibition of PT extension but did not affect growth polarity or germination rates	Guo et al., 2013
***Oryza sativa***					
*Os*CPK25	Os11g04170	-	-	-	Wang et al., 2011; Tang and Page, 2013
*Os*CPK26	Os12g03970	-	-	-	Wang et al., 2011; Tang and Page, 2013

a Short name.

b Gene identifier according to TAIR (*A. thaliana* CDPKs) or GeneBank (other species).

c Subcellular localization published in the literature: S, soluble; MB, membranes; N, nucleus; C, cytoplasm; P, peroxisomes; PM, plasma membrane; ER, endoplasmic reticulum.

d Lists of published CPK target genes with their published subcellular localization in parentheses.

e Physiological relevance is defined by phenotypes of knockdown or overexpressing lines, when available, or other physiological traits.

f Corresponding references.

## Crosstalk with Other Signaling Networks in Pollen Tube Growth

Certainly, proper growth of the PT depends on an elaborate mechanism, which not only needs the central Ca^2+^/CPK signal but also needs integration and coordination with other molecules and signaling systems, such as ROP1 signaling, inositol-polyphosphates (IP3/6) and numerous pistil factors (γ-aminobutyric acid, long-chain base phosphates, and polyamines; Wu et al., 2014; Yu et al., 2014; Aloisi et al., 2017; Domingos et al., 2019). Additionally, some evidence reveals a link between [Ca^2+^]_cyt_ and pH_cyt_ plays a role in PT growth (Behera et al., 2018; Mangano et al., 2018). Further, we will emphasize some interconnections and convergence points of Ca^2+^ signaling with ROS and adenosine 3',5'-cyclic monophosphate (cAMP). ROS generated by NADPH oxidases (NOXs) that are shown to be involved in various processes in PT growth, including germination, polarized, and ovule-targeted growth, and PT burst during fertilization (see review by Wudick and Feijo, 2014). Some direct evidence indicates that binding of Ca^2+^ will activate some NOXs activities, such as RbohH and RbohJ (Potocky et al., 2012; Kaya et al., 2014). This activation mechanism probably occurs synergistically with phosphorylation of NOXs, although phosphorylation seems to be a prerequisite for Ca^2+^-mediated NOX activation (Kimura et al., 2012). Based on these findings, a positive feedback model for Ca^2+^/ROS signaling in PT growth is raised, in which Ca^2+^-induced NOXs activity leads to ROS mediated activation of some Ca^2+^ channels, which in turn causes an increase in the cytosolic Ca^2+^ level (Breygina et al., 2016; Makavitskaya et al., 2018). An interesting recent finding is that LINC-complex mediated VN proximity to the PT tip is required for both responses to exogenous ROS and internal nuclear Ca^2+^ fluctuations (Moser et al., 2020). Cyclic nucleotides (cNMPs), such as cAMP and guanosine 3',5'-cyclic monophosphate (cGMP), as the activators of CNGCs are undoubtedly involved in PT growth (Duszyn et al., 2019). Presently, information is limited because there are only seven experimentally confirmed adenylate cyclases (ACs) in higher plants, which limits the knowledge about how cAMPs were synthesized and how they regulate the CNGCs during PT growth (Yang et al., 2020). Among them, *Zm*PSiP is preferentially expressed in PT and catalyzes the production of cAMP, which is responsible for PT growth and reorientation (Moutinho et al., 2001). As for cGMP, it is noteworthy that nitric oxide can activate guanylyl cyclase and possibly activate CNGCs through increases in cGMP levels, leading to an influx of extracellular Ca^2+^ and actin filament organization during cell wall construction in *Pinus bungeana* PTs (Wang et al., 2009; Marondedze et al., 2017). Therefore, capturing a more complete picture of the Ca^2+^/CPK signaling in PT growth requires an exhaustive investigation of the other integrated molecular and signaling systems.

## Concluding Remarks and Future Perspectives

Although substantial progress has been made in the past decades, the mechanism of the Ca^2+^/CPKs signal pathway for regulating PT growth is still fragmented. Only a few relatively complete signal transduction chains are reported. Besides the regulation of the cell wall properties and ion concentrations, the related researches about the Ca^2+^/CPKs signal pathway involved in other processes such as endo- and exo-cytosis and cytoskeletal regulation fine-tuning of Ca^2+^ concentration in organelles (vacuole, dictyosome, and mitochondria) need exhaustive investigation (Steinhorst et al., 2015; Selles et al., 2018; Flores-Herrera et al., 2019; Guo and Yang, 2020). Moreover, identification of the unknown targets of CPKs (particularly for nuclear targets such as TFs) and depiction of the elaborate internetwork of the Ca^2+^/CPKs pathway with other signal pathways will lead to important insights into the mechanisms of PT growth. The progress of experimental techniques such as various omics techniques, Y2H screens, CRISPR/Cas gene editing, and RNAi by directly adding the siRNAs into the PT culture medium (Suwinska et al., 2017), various molecular probes (Mravec et al., 2017), microfluidics and microrobotics (Burri et al., 2020), and computational methods (Damineli et al., 2017) will provide new opportunities and boost our understanding of the Ca^2+^/CPKs signal pathway in PT growth.

## Author Contributions

HY, CY, SY, and YZ wrote the manuscript. FY, NC, XL, YL, and XH revised and critically evaluated the manuscript. All authors contributed to the article and approved the submitted version.

### Conflict of Interest

The authors declare that the research was conducted in the absence of any commercial or financial relationships that could be construed as a potential conflict of interest.
